# Global, regional, and national burden of chronic kidney disease, 1990-2021: a systematic analysis for the global burden of disease study 2021

**DOI:** 10.3389/fendo.2025.1526482

**Published:** 2025-03-05

**Authors:** Kaifeng Xie, Haihong Cao, Shiyun Ling, Jiameng Zhong, Haitao Chen, Penghui Chen, Renfa Huang

**Affiliations:** Nephropathy Department, Shenzhen Hospital (Futian) of Guangzhou University of Chinese Medicine, Shenzhen, China

**Keywords:** chronic kidney disease, disability-adjusted life-years, join-point regression model, disease burden, GBD 2021

## Abstract

**Background:**

Chronic kidney disease (CKD) continues to represent a significant public health concern, with both prevalence and incidence rates on the rise globally. Therefore, the study employed the Global Burden of Disease (GBD) database to investigate the global burden of CKD from 1990 to 2021.

**Methods:**

This study utilized data from the GBD 2021. Join-point regression models were developed for the estimation of the average annual percentage change (AAPC) in the prevalence and mortality rates of CKD. Subsequently, stepwise multiple linear regression analysis was conducted to examine the trends in disability adjusted life years (DALYs) and DALYs rate for CKD across diverse populations between 1990 and 2021. Moreover, the influence of age, gender, and socio-demographic index (SDI) on the burden of CKD among patients from 1990 to 2021 was examined. Furthermore, the projection of the burden of CKD from 2022 to 2032 was also conducted.

**Results:**

The AAPC for prevalence and mortality rates across the entire period spanning 1990 to 2021 was 0.92 and 2.66, respectively. A notable increase in the DALYs and DALYs rate for CKD was demonstrated over time, indicating a growing CKD burden on society since 1990. Furthermore, the DALYs rates for CKD were lowest in the 5-9 year age group for both genders, rising thereafter with age. Notably, the DALYs rate for CKD was higher in males than in females. Regions with higher SDI, generally exhibited a lower burden of CKD, while less developed regions, demonstrated the opposite pattern. Additionally, the age-standardized prevalence and mortality rates for CKD would be projected to increase to 8,773.85 and 21.26 per 100,000 individuals, respectively, by 2032.

**Conclusion:**

The research indicated a gradual increase in the global prevalence and mortality rates of CKD over time, which might prompt the formulation of more efficient health policies to alleviate its burden.

## 1 Introduction

Chronic kidney disease (CKD) is recognized as a significant and pressing global public health issue. Owing to the global growth and aging of the population, the prevalence and incidence of CKD have risen by 40% over the past three decades (1, 2). CKD results from multiple causes and is characterized by abnormalities in kidney structure or function, which persist for at least three months, leading to adverse health outcomes (3). According to the 2012 KDIGO guidelines, the diagnostic threshold for CKD is defined as an estimated glomerular filtration rate (eGFR) of less than 60 mL/min/1.73 m² and an albumin-creatinine ratio of 30 mg/g or higher (4). The ultimate progression of CKD is end-stage renal disease (ESRD), a severe complication requiring renal replacement therapy (peritoneal dialysis, hemodialysis, or kidney transplantation). This condition is associated with numerous adverse outcomes, including increased risks of cardiovascular disease (CVD), mortality, and infection (5). Early and accurate diagnosis, along with appropriate treatment of CKD, can significantly reduce patient mortality and prevent the disease’s progression and the onset of complications. Consequently, it is imperative to investigate further the factors influencing CKD’s burden to enable more countries to recognize its significant impact on global health. Such understanding could help guide efforts to enhance public awareness, develop clinical prevention and intervention strategies, and establish comprehensive care for CKD patients.

The Global Burden of Disease (GBD) database, organized by leading global health research institutions, serves to assess and analyze the health impacts of diseases, injuries, and risk factors worldwide and across different regions. This database represents the largest and most comprehensive scientific study to date, aimed at quantifying global human health levels and trends (6). The disability-adjusted life year (DALY) quantifies disease burden, while DALY reduction indicates a decrease in years of life lost (YLLs) or years lived with disability (YLDs) (7). Previous studies from the GBD indicate that the CKD-DALY ranking has increased from the 29th in 1990 to the 18th position in 2019 (8). A thorough understanding of CKD’s global impact is essential to optimize the allocation of medical resources across various countries and regions. However, assessments and analyses of the global, regional, and national CKD burden currently rely on the GBD 2019 database. Liu et al., based on their analysis of the 2019 GBD database, found that the global burden of hypertension-related CKD is increasing (9). However, the most recent GBD 2021 data has yet to be utilized for a comprehensive analysis of this burden.

Consequently, based on the GBD 2021 data, this study conducted an analysis of the epidemiological characteristics of CKD across 204 countries and regions for the period 1990 to 2021 and examined the correlations between these characteristics and factors such as age, gender, and the socio-demographic index (SDI). Furthermore, this study leveraged historical data to forecast the global CKD burden for the upcoming decade, from 2022 to 2032, aiming to inform policy-making and improve epidemiological surveillance of CKD. These insights will be expected to be invaluable to medical professionals and policymakers, facilitating informed decision-making and the development of effective CKD management strategies.

## 2 Materials and methods

### 2.1 Data acquisition

The GBD (https://ghdx.healthdata.org/ihme_data) 2021 database conducted a comprehensive evaluation of the global burden of diseases, injuries, and risk factors across various age and gender groups. The database comprised data on 371 diseases or injuries and 88 risk factors, spanning the period from 1990 to 2021, and encompassing 204 countries and territories (1). The GBD 2021 project undertook an assessment of the rates, counts, and percentage variations in prevalence, deaths, and DALYs for a range of diseases. The information pertaining to the aforementioned estimated indices was provided in a comprehensive manner within the appendix of the GBD 2021 capstone paper (10).

The classification of CKD adhered to the International Classification of Diseases, 10th Revision (ICD-10). The ICD-10 codes pertinent to CKD included D63.1, E10.2, E11.2, E12.2, E13.2, E14.2, I12-I13.9, N02-N08.8, N15.0, N18-N18.9, and Q61-Q62.8 (2). Subsequently, patients with the relevant codes pertaining to CKD were included from the GBD database by accessing the GBD Results Tool (https://vizhub.healthdata.org/gbd-results/) (access time: 21st October 2024). Moreover, the raw data on CKD, spanning from 1990 to 2021, was downloaded and encompassed five subtypes: CKD was attributed to a number of different aetiologies, including type 1 and type 2 diabetes, hypertension, glomerulonephritis, and other causes.

### 2.2 SDI

The SDI was a measure of a country or region’s level of development. The SDI values, with a range of 0 to 1, were employed as an indicator of a country’s overall fertility rate among women under the age of 25, educational attainment levels and the distribution of lower per capita income. Based on SDI values in GBD 2021, the countries and territories were categorized into five development levels: low, low-middle, middle, high-middle, and high.

### 2.3 Statistical analysis

The glmnet package (v 4.1-2) (11) was employed to construct a join-point regression model. Minutely, the temporal characteristics of the CKD distribution were used to inform the construction of a piecewise regression model, which was then employed to perform trend fitting and optimization on the data points within each segment. The annual percentage change (APC) and AAPC of the model were calculated. APC evaluated the trend within individual segments of the segmented function, whereas AAPC assessed the overall average trend across the fixed intervals. Subsequently, the analysis incorporated the variable of gender, and the ggplot2 package (v 3.4.3) (12) was used to generate line graphs depicting the prevalence and mortality rates of CKD across different genders from 1990 to 2021. Furthermore, a join-point regression model was employed to elucidate the trends in prevalence and mortality rates of CKD over the same period. The glmnet package (v 4.1-2) was used to conduct a stepwise multiple linear regression analysis, examining the trends in DALYs and DALYs rate of CKD among various populations from 1990 to 2021. The data were subjected to statistical analysis using a 95% confidence interval (CI). The results were illustrated using smooth curves plotted with the ggplot2 package (v 3.4.3). Furthermore, the impact of both age and gender on the burden of CKD among patients in 1990 and 2021 was examined. This study covered an age range of 0-89 years, divided into 18 groups, each spanning five years. Histograms were generated using the ggplot2 package (v 3.4.3) to facilitate the comparison of differences and trends in DALYs and DALYs rate from 1990 to 2021, across different genders and age groups. Additionally, the ggplot2 package (v 3.4.3) was employed to demonstrate the geographical distribution of the burden of CKD across different country regions, with a particular focus on identifying disparities through the use of DALYs, DALYs rate, and age-standardized DALYs rate (ASDR). The ggplot2 package (v 3.4.3) was employed to generate histogram and stacked plot, which were used to illustrate the ASDR for populations of different genders across various regions, together with the total DALYs cases for those populations from 1990 to 2021. Moreover, the generation of smooth curves was achieved through the utilization of the ggplot2 package (v 3.4.3), with the objective of depicting the trend of SDI in relation to ASDR of varying country regions from 1990 to 2021. The aforementioned curves were accompanied by correlations calculated using the Spearman method. In addition, the forecast package (v 8.23.0) (https://pkg.robjhyndman.com/forecast/) and tseries package (v 0.10-58) (https://CRAN.R-project.org/package=tseries) were employed in the construction of the autoregressive integrated moving average (ARIMA) model, and the model was evaluated through a white noise test. ARIMA’s prediction of variable changes over a given future time range is primarily based on the following assumptions:1. Stationarity assumption: The ARIMA model assumes that the time series is stationary, meaning that its statistical properties (such as mean and variance) remain constant over time. If the data is non-stationary, it is typically transformed into a stationary series through differencing.2. Linear relationship assumption: The model assumes a linear relationship between the current and past values in the time series, which is captured by the autoregressive (AR) and moving average (MA) components.3. White noise residual assumption: The model assumes that the residuals (i.e., prediction errors) follow a white noise process, meaning they have a mean of zero, constant variance, and are uncorrelated at different time points. If residuals exhibit autocorrelation, it indicates that the model requires further refinement.4. Limited historical impact assumption: In the MA component, the model assumes that only the past q error terms influence the current value, while more distant error terms have no direct effect. The auto.arima function from the forecast package (v 8.23.0) was employed to identify the optimized ARIMA model that minimizes both the akaike information criterion (AIC) and the bayes information criterion (BIC). Subsequently, the optimized model was employed to predict the prevalence and mortality rates of CKD over the subsequent ten-year period, with statistical analysis incorporating 95% CI. The results of the prediction were presented using the ggplot2 package (v 3.4.3). Glmnet: This package is used to implement generalized linear models (GLM) and supports regularization (such as LASSO and Ridge regression). It is often used to model high-dimensional data.Ggplot2: This is a package for data visualization that provides powerful drawing functions, especially suitable for creating complex graphics, such as scatter plots, box plots, etc. Statistical analyses were conducted utilizing the R language (v 4.2.2), and the results were considered statistically significant if the associated P < 0.05.

## 3 Results

### 3.1 Trends in the global disease burden of CKD

**Figure 1:**
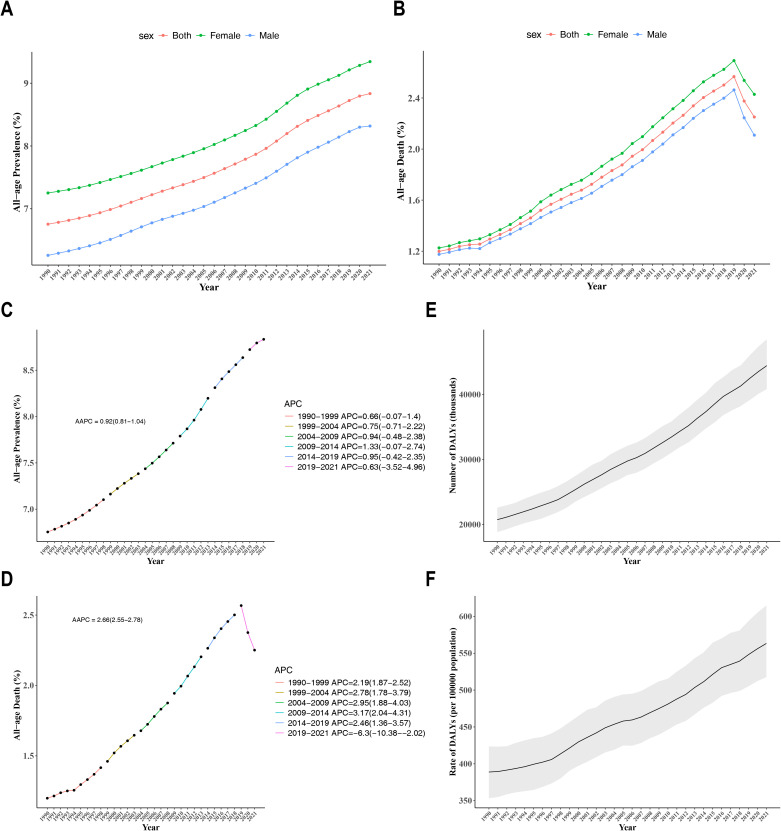
**(A)** Temporal trends of prevalence attributable to CKD in males and females from 1990 to 2021; **(B)** Temporal trends of mortality e attributable to CKD in males and females from 1990 to 2021; **(C)** Joinpoint regression model for the prevalence of CKD from 1990 to 2021; **(D)** Joinpoint regression model for CKD mortality from 1990 to 2021; **(E)** Trends in DALYs for CKD from 1990 to 2021; **(F)** Trends in DALY rates for CKD from 1990 to 2021.

Globally, the prevalence rate of CKD across all-age and both genders exhibited an upward trend from 1990 to 2021 (AAPC = 0.92, 95% CI: 0.81 to 1.04) (**Figure 1A**). The mortality rate among different gender populations with CKD increased from 1990 to 2019, but subsequently exhibited a notable decline after 2019 (AAPC = 2.66, 95% CI: 2.55 to 2.78) (**Figure 1B**). Notably, female patients with CKD exhibit higher morbidity and mortality rates than male patients. Furthermore, join-point analysis was employed to identify the segmental trends in the prevalence and mortality rates of CKD. Since 1990, the prevalence rate of CKD has significantly risen, with a particularly pronounced surge occurring between 2009 and 2014 (APC = 1.33, 95% CI: -0.07 to 2.74) (**Figure 1C**). In 2019, the trend of CKD mortality rate underwent a reversal, with APCs of 2.46 (95% CI: 1.36-3.57) from 2014 to 2019 and -6.3 (95% CI: -10.38 to -2.02) from 2019 to 2021 (**Figure 1D**). The DALYs for CKD and the DALYs rate had demonstrated a notable increase over time, indicating an escalating disease burden on society since 1990 (**Figures 1E, F**). By 2021, the burden of CKD had nearly doubled in comparison to 1990.

### 3.2 Global disease burden of CKD by age and gender

**Figure 2:**
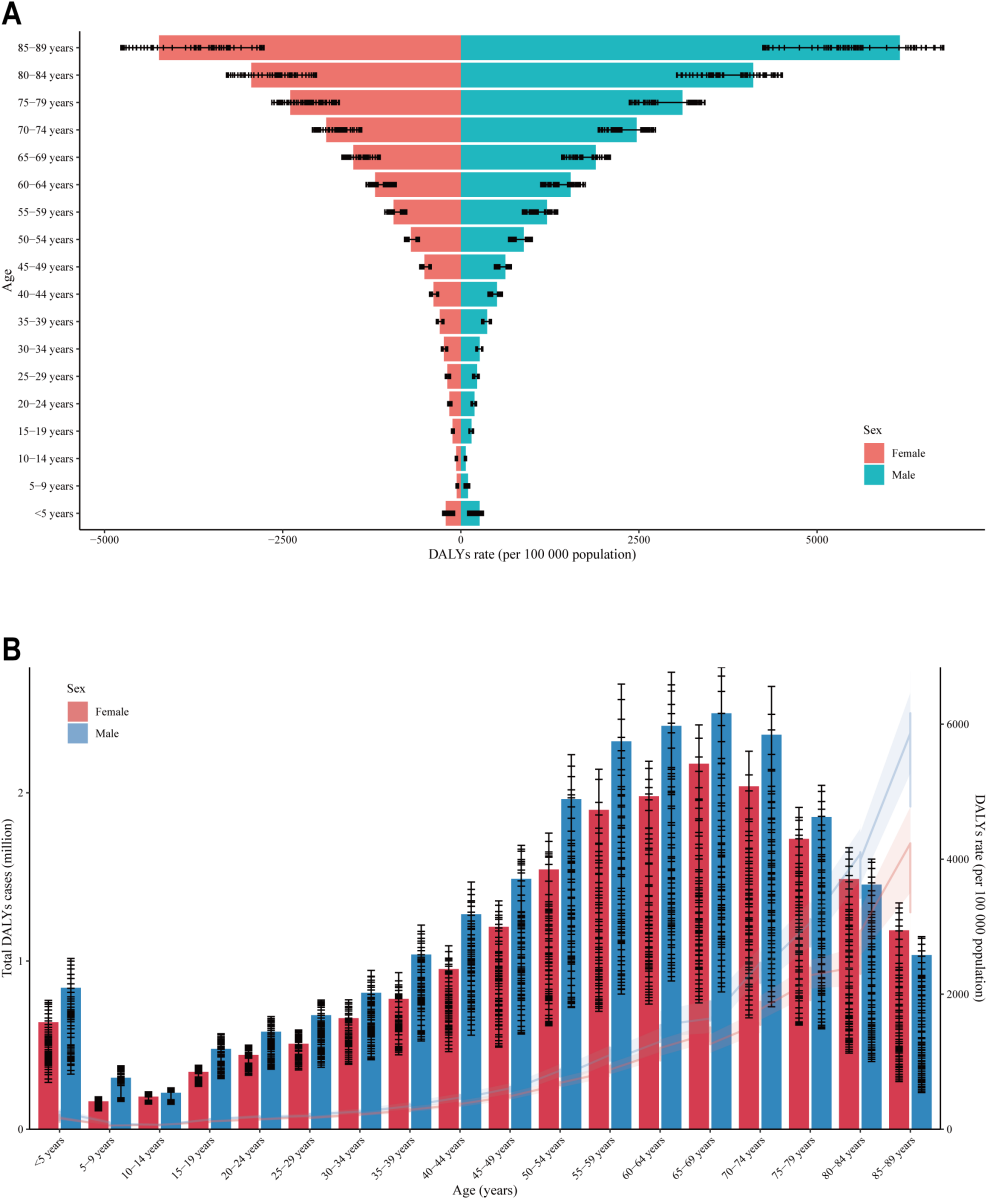
Distribution of DALY rates across different age groups and between genders. **(A)** The horizontal axis is the DALYs rate, and the vertical axis is the age segment; **(B)** The vertical axis is the DALYs rate, and the horizontal axis is the age segment; DALY (Disability-Adjusted Life Year) is an indicator used to measure the health burden. The DALY rate refers to the number of disability-adjusted life years caused by a specific disease, injury, or health issue per unit population. A higher DALY rate indicates a heavier health burden on the region or population. The range of 0-5000 represents the male DALY rate, while the range of -5000 to 0 represents the female DALY rate.

The lowest DALYs rate for CKD was observed in the 5-9 year age group for both genders, with rates increasing with age thereafter (**Figure 2A**). Remarkably, males exhibited a higher DALYs rate of CKD compared to females. In accordance with the aforementioned, the total DALYs cases for CKD were observed to be the lowest in the 5-9 year age group for both genders (**Figure 2B**). Thereafter, an increase in total DALYs cases was observed with age, reaching a peak in the 65-69 age group, followed by a slight decline. It was observed that, with the exception of the 80-84 age group and the 85-89 age group, males exhibited a higher total DALYs cases than females across the majority of age groups.

### 3.3 Distribution disparity in the burden of CKD

**Figure 3:**
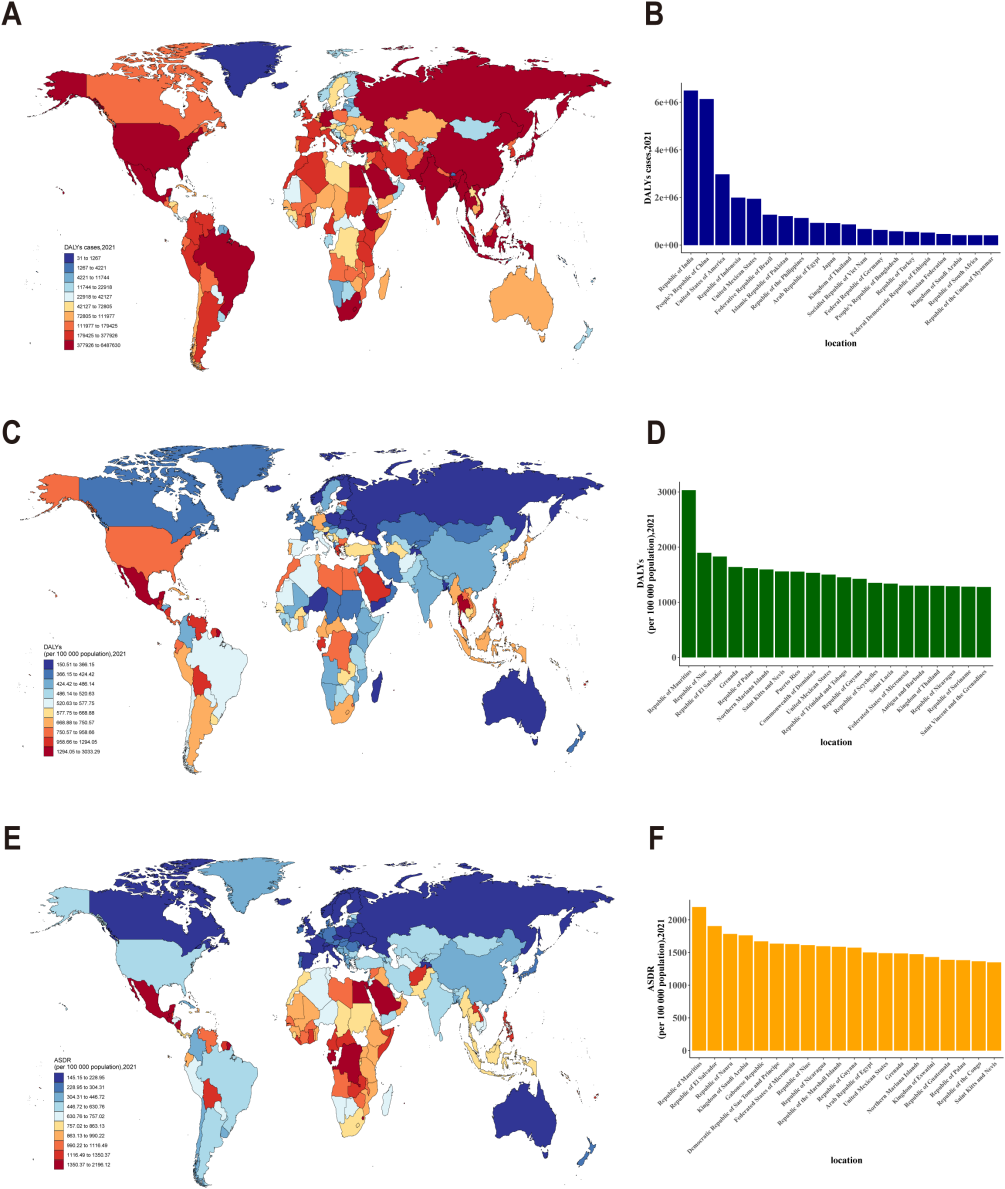
**(A)** Distribution of DALYs cases due to CKD across different countries and regions; **(B)** DALYs cases of CKD in different countries and regions (top 20); **(C)** Distribution of DALY rates for CKD across different countries and regions; **(D)** DALY rates for CKD in different countries and regions (top 20);**(E)** Age-standardized DALY rates(ASDR) for CKD across different countries and regions; **(F)** ASDR for CKD in different countries and regions (top 20).

Significant discrepancies were evident in DALYs, DALYs rate, and ASDR for CKD across diverse countries and regions. Specifically, India and China exhibited the highest cases of DALYs attributable to CKD in 2021 (**Figures 3A, B**). After adjusting for population size, Mauritius exhibited the highest rate of DALYs, specifically exceeding 3,000 DALYs per 100,000 population for CKD (**Figures 3C, D**). Interestingly, after accounting for population size and age, Mauritius continued to exhibit the highest burden, with an ASDR exceeding 2,000 per 100,000 population (**Figures 3E, F**). The results indicated considerable discrepancies in the global burden of CKD across countries and regions, with particularly elevated rates observed in specific areas, such as Mauritius.

### 3.4 Impact of SDI on the burden of CKD

**Figure 4:**
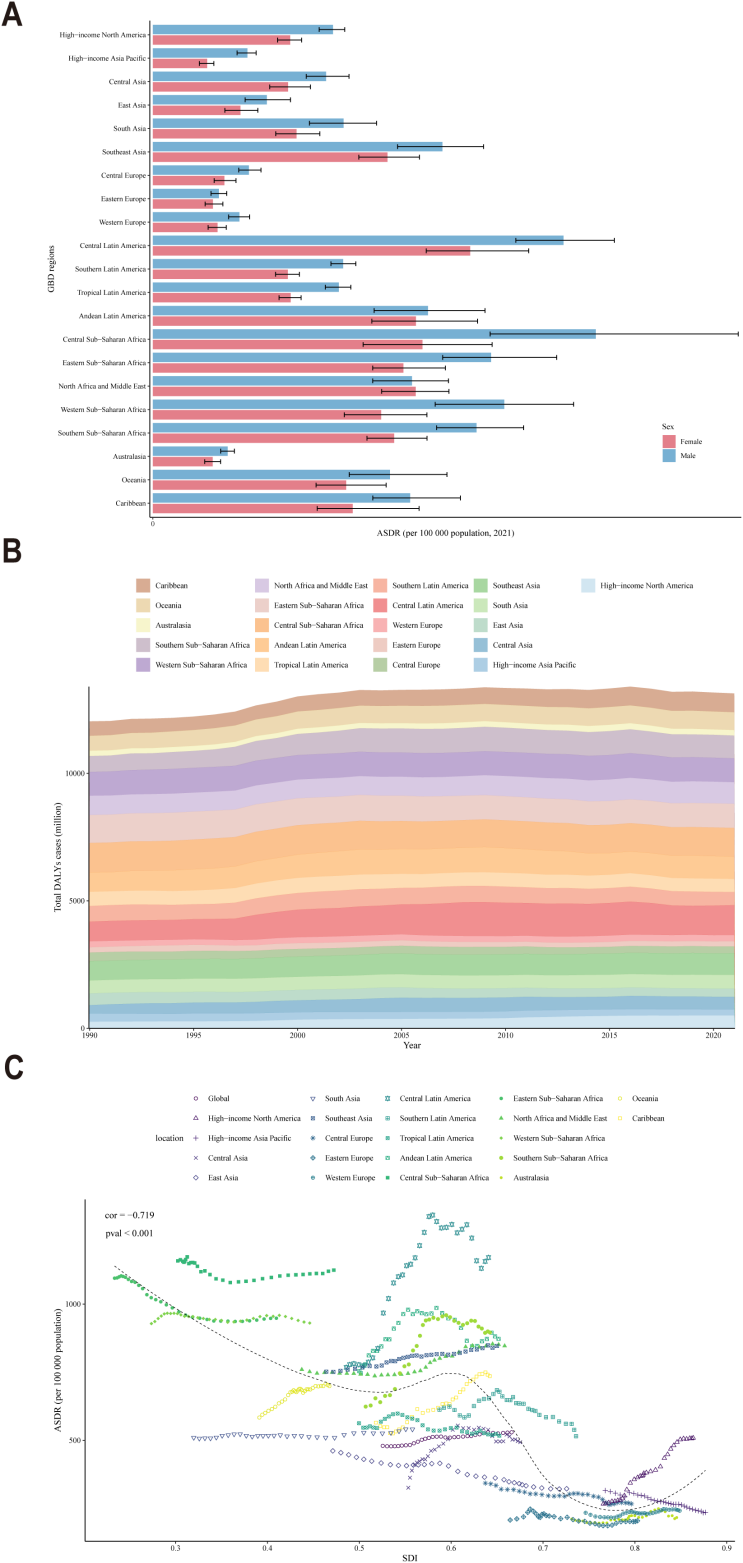
**(A)** ASDR distribution by gender across various countries and regions in 2021; **(B)** DALYs cases across all ages in various countries and regions from 1990 to 2021; **(C)** Trends in SDI and ASDR across all age groups in various countries and regions from 1990 to 2021.

Analyses based on the SDI classification regions in 2021 revealed that regions with a high SDI, such as Central Europe, Eastern Europe, Western Europe and Australasia, exhibited a lower ASDR, indicating a lower CKD burden (**Figure 4A**). In contrast, regions with a low SDI, such as Central Sub-Saharan Africa and Central Latin America, exhibited relatively higher ASDR, which correspond to a greater burden of CKD. Moreover, the burden of CKD was consistently higher among males compared to females across all regions, with a narrower gender gap observed in developed regions compared to less developed ones. The total DALYs cases for populations across various regions from 1990 to 2021 served to reinforce the conclusion that the CKD burden was more significant in low-SDI regions and relatively lower in high-SDI regions (**Figure 4B**). Besides, a significant negative correlation was observed between SDI and ASDR across global, regional, and national populations from 1990 to 2021 (correlation coefficient (cor) = -0.719, P < 0.001) (**Figure 4C**). This indicated that developed regions, such as Europe, with high SDI, generally exhibited low ASDR, whereas less developed regions, such as Africa, demonstrated the opposite trend.

### 3.5 Prediction of CKD prevalence and mortality rates in the next decade

**Figure 5:**
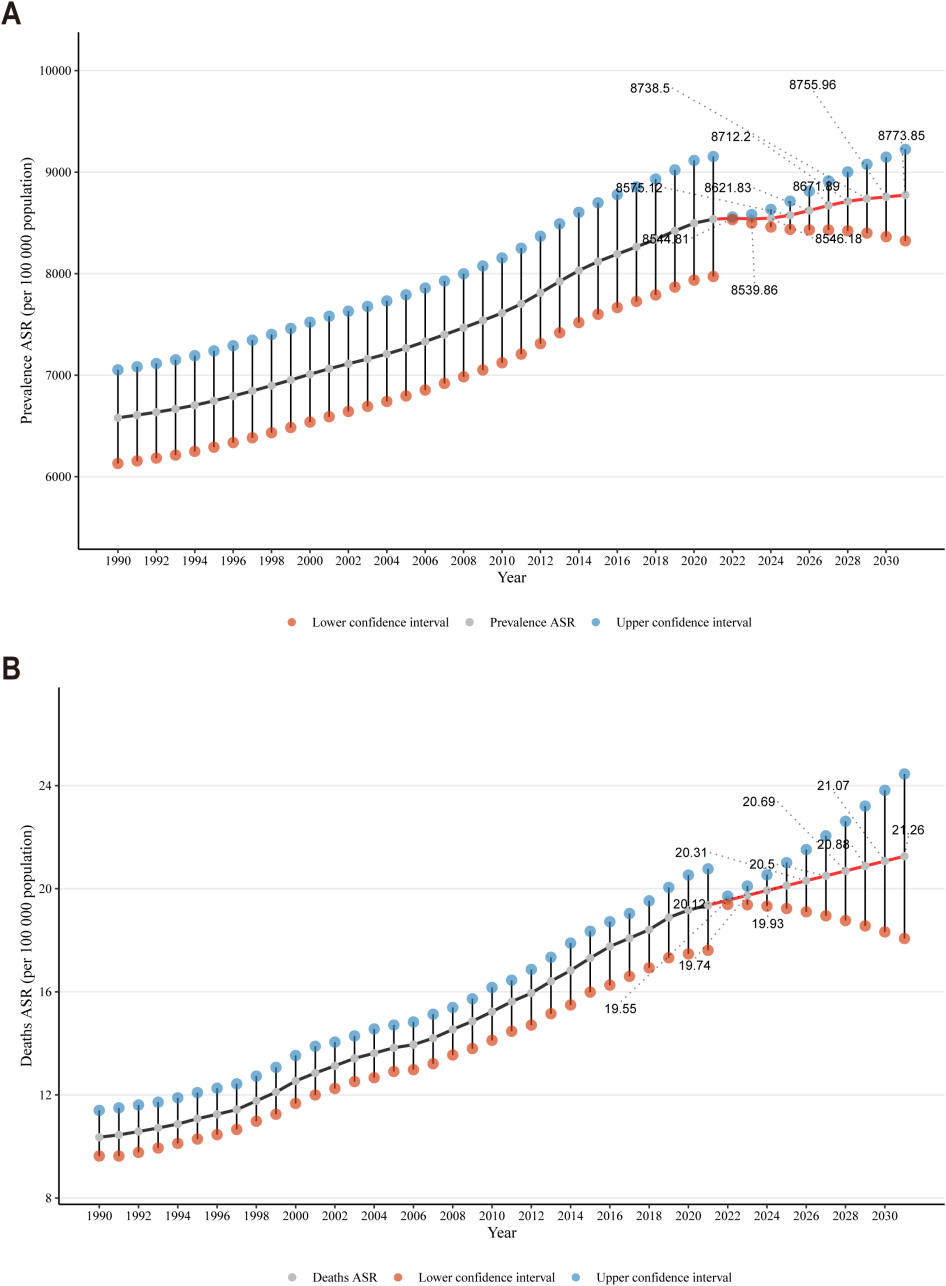
**(A)** Temporal trends in CKD prevalence projections over the next decade; **(B)** Temporal trends in CKD mortality projections over the next decade.

The ARIMA model was employed to quantify the projected trends in CKD prevalence and mortality rates over the subsequent ten-year period. Between 1990 and 2021, the age-standardized prevalence rate of CKD exhibited an upward trajectory, followed by a decline in 2022 and a gradual increase thereafter until 2032 (**Figure 5A**). Specifically, the rate was projected to increase from 8,544.81 per 100,000 population in 2022 to 8,773.85 per 100,000 population in 2032. The age-standardized mortality rate for CKD exhibited an upward trajectory from 1990 to 2021, a trend that persisted beyond the latter date (**Figure 5B**). Projections indicated that the rate was likely to increase from 19.55 per 100,000 population in 2022 to 21.26 per 100,000 population in 2032. The results indicated an upward trajectory in the incidence and mortality of CKD over the forthcoming decade, which would present a substantial challenge to global health.

## 4 Discussion

CKD is a common non-communicable disease that affected more than 650 million people worldwide and caused more than 1.2 million deaths in 2017 (13). Currently, as the prevalence of diabetes, hypertension, and metabolic syndrome increases among the elderly, the incidence of kidney disease is expected to rise, potentially making CKD the fifth leading cause of death globally by 2040 (14). This study examined the global, regional, and national burden of CKD from 1990 to 2021, revealing that both prevalence and mortality rates have been on the rise, with this upward trend anticipated to persist beyond 2022. Additionally, these findings indicate that the DALYs rate for CKD was lowest among the 5-9 age group, increasing progressively with age. Men bear a greater burden of CKD compared to women; regions with a higher SDI report a lighter CKD burden, whereas less developed regions exhibit a heavier one. These findings demonstrate that age, gender, and socioeconomic development significantly influence the distribution and severity of CKD. This provides a new reference for improving the means of CKD prevention, enhancing early control and enriching treatment strategies.

Over the past two decades, most countries and regions have witnessed rapid population growth along with ongoing urbanization and industrialization. These factors have collectively contributed to an escalating burden of CKD, marked by rising numbers of deaths and DALY cases attributed to CKD (15). While the DALY rate from non-communicable diseases has been declining, this positive trend has not been observed in CKD (16). These findings indicate that the prevalence of CKD across all age groups and genders has consistently increased each year from 1990 to 2021.Concurrently, the mortality rate also demonstrated an overall upward trend throughout this period. However, notably, after 2019, the mortality rate began to exhibit a distinct downward trend. Given the severe impact of the COVID-19 pandemic since 2019, patients with CKD are at an increased risk of contracting COVID-19 (17). Research has confirmed that CKD is linked with more severe SARS-COV-2 infections among various comorbidities, especially in patients undergoing renal replacement therapy (18). Consequently, the observed decrease in CKD mortality since 2019 may not reflect a true reduction in the number of deaths from CKD. Instead, many patients with CKD or undergoing renal replacement therapy may have died from various complications during the COVID-19 pandemic, which were not classified as CKD-related deaths. Therefore, future studies should more clearly differentiate the true impact of COVID-19 on CKD outcomes and mortality statistics. Long-term follow-up studies, stratified analyses, multicenter comparisons, statistical modeling, and data sharing can enable researchers to more precisely elucidate the true impact of COVID-19 on CKD patient mortality. Additionally, both DALYs and DALY rates for CKD have progressively increased over time, signifying a growing societal burden of the disease since 1990. Specifically, from 1990 to 2016, CKD incidence rose by 89%, prevalence by 87%, CKD-DALYs by 62% globally (19). The ongoing increase in the global CKD burden is likely influenced by various regional factors (e.g., population aging, dietary habits, obesity rates) and policy drivers (e.g., public health policies, health education, and chronic disease management) (20). As the global aging population continues to rise, particularly in high-income countries, the prevalence of CKD among the elderly has markedly increased. Additionally, changes in modern dietary patterns, particularly the heightened consumption of high-salt, high-sugar, and high-fat foods, have become significant contributors to the growing prevalence of CKD (21). Dietary interventions are crucial in managing chronic diseases such as hypertension, diabetes, and obesity, all of which are strongly associated with the onset and progression of CKD (22). Studies have shown that dietary interventions, particularly low-sodium and low-protein diets, can effectively reduce blood pressure, control blood sugar, and support weight management, thereby providing indirect protection to the kidneys (23). The rising obesity rates are a key factor driving the global increase in the burden of CKD (24). In low- and middle-income countries, the lack of effective public health policies and early CKD screening programs is a significant factor contributing to the increasing burden of CKD.

Previous epidemiological data have demonstrated that age is an independent and significant risk factor for CKD, with mortality rates due to renal impairment varying across different age groups (25). Our study integrated age, gender, and the SDI to analyze and explore the impact of the CKD burden. The results suggested that the DALY rate for CKD was lowest in the 5-9 age group, increased progressively with age, peaked in the 65-69 age group, and then declined slightly. It is important to note that using a uniform eGFR threshold as a diagnostic criterion for CKD across all age groups may lead to misdiagnosis. This could result in many elderly individuals, who experience normal age-related eGFR decline, being misclassified as having CKD (26). Future revisions of CKD guidelines should consider adjusting the diagnostic criteria, particularly the eGFR thresholds for different age groups (27, 28). In conjunction with previous epidemiological data and clinical experience, elderly individuals are more likely to suffer from underlying conditions such as hypertension, diabetes, and cardiovascular disease, which increases their risk of CKD (29, 30). Cardiovascular diseases exacerbate the burden on the kidneys and impair renal tubular function through mechanisms such as hypertension and arteriosclerosis. Metabolic syndrome, which includes conditions like diabetes, obesity, and lipid abnormalities, induces insulin resistance and fat deposition through metabolic disruptions, thereby worsening renal inflammation and promoting fibrosis. Chronic inflammation activates immune responses via pro-inflammatory factors (such as TNF-α and IL-6), damaging renal tubules and glomeruli and accelerating renal function decline. The synergistic effect of these factors accelerates the progression of CKD (31–33). The disparities in CKD prevalence across regions are closely linked to comorbid factors such as cardiovascular disease, metabolic syndrome, and chronic inflammation. Particularly in low-income countries or specific Asian populations, the high prevalence of cardiovascular disease and metabolic syndrome may further exacerbate the burden of CKD (34). Hypertension and diabetes are widely recognized as major risk factors for CKD (35). These chronic diseases damage the kidneys through multiple mechanisms. Elevated blood sugar and high blood pressure accelerate injury to renal microvessels, impair the normal function of renal tubules and glomeruli, and ultimately lead to a gradual decline in renal function (36). In the 65-69 age group, many individuals suffer from long-term conditions such as hypertension and diabetes, and managing these conditions becomes increasingly challenging with age (37). As individuals age, the function of renal tubules and glomeruli naturally declines, and the kidneys’ ability to tolerate damage diminishes. In elderly individuals with underlying conditions like hypertension and diabetes, the rate of renal function decline may accelerate, leading to a higher burden of disease (38), as reflected in the increased DALY in this age group.CKD complications are more prevalent and severe in elderly patients, negatively impacting their prognosis (39). Therefore, early diagnosis and targeted treatment of CKD are crucial for its prevention and management, with particular attention given to the risk of CKD in middle-aged and elderly populations. For young individuals, particularly those aged 20-40, early detection and management of underlying conditions like hypertension and diabetes can significantly mitigate their long-term impact on kidney health, thereby reducing the future risk of CKD (40). Maintaining normal blood sugar and blood pressure levels can significantly reduce the risk of CKD. Therefore, early intervention in younger populations is crucial, as it helps control the progression of chronic diseases and greatly lowers the incidence and burden of CKD.

Although diabetes and hypertension are the primary known risk factors for CKD, obesity, environmental exposure, and genetic susceptibility have emerged as significant contributors to CKD in recent years. Unfortunately, the GBD database does not include data on factors like obesity, environmental exposure, and genetic susceptibility, making it difficult to analyze their correlation with CKD through this database. Research has shown that obesity is a key driver of CKD progression, with mechanisms including hemodynamic changes, inflammation, oxidative stress, and activation of the renin-angiotensin-aldosterone system (RAAS) (41). A cohort study in the UK found that overweight and obesity, even without metabolic abnormalities, were associated with a higher risk of developing CKD compared to individuals with normal weight and no metabolic abnormalities (42). Environmental factors, such as air pollution, heavy metal contamination, and water pollution, are also considered potential triggers for chronic diseases (43). Studies have suggested that exposure to air pollution may play a crucial role in the incidence and progression of hypertension, diabetes, and CKD (44). Furthermore, genetic factors are pivotal in the development of CKD (45). For example, APOL1 gene variations have been strongly linked to the high incidence of CKD in African-American populations (46). Future research could focus on how genetic screening might be used to identify high-risk populations and develop personalized interventions based on genetic profiles.

The global burden of CKD varies by sex and age, with this results indicating that men experience a significantly greater burden than women. Men exhibit higher DALY rates than women across most age groups, except in the 80-89 age group where women exhibit higher DALY rates. Differences in physiological structure and hormone levels between men and women may contribute to the differing burdens of CKD.Studies suggest that estrogen may protect women, whereas testosterone and unhealthy lifestyles contribute to a more rapid decline in renal function among men (47). Estrogen indirectly reduces the incidence of CKD by lowering the risk of cardiovascular disease; however, after menopause, a decline in estrogen levels may increase women’s risk of CKD (48). Thus, sex hormone differences may underlie the biological basis for sex differences in CKD. Additionally, lifestyle factors such as smoking, alcohol consumption, unhealthy eating habits, and a greater tendency to neglect health assessments are more prevalent in men, contributing significantly to the heavier CKD burden in this population. While CKD is globally more prevalent among women, end-stage renal failure occurs more frequently in men, particularly those undergoing renal replacement therapy (49). To address the differences in health behaviors between men and women, we recommend designing targeted health education programs, particularly to raise men’s awareness of early screening and CKD management. Given the differences between men and women in physiology, behavior, and social roles, adopting personalized strategies for the management and treatment of CKD is recommended. This study utilizes DALY, DALY rate, and ASDR to quantify the CKD burden across various countries and regions.

Research from China indicates that, from 1990 to 2019, CKD prevalence increased from 6.7% to 10.6%, and the mortality rate rose from 8.3 per 100,000 to 13.8 per 100,000 (2). A recent study analyzing CKD prevalence in 161 countries reported a global median prevalence of 9.5% (IQR 5.9-11.7), with Eastern and Central Europe showing the highest rates (12.8%, 11.9-14.1) (50). Findings align with prior GBD studies, and the consistently increasing DALY rates underscore a changing burden of CKD, highlighting the ongoing need for a global focus on prevention and long-term management strategies.

Notably, Mauritius exhibits significant regional variations in the burden of CKD, particularly in terms of the highest DALYs per 100,000 population. Mauritius is a multi-ethnic country, with a population primarily composed of Indian, African, and European ethnic groups, each of which may have varying susceptibilities to chronic diseases. Additionally, the traditional Mauritian diet is typically high in salt, sugar, and fat, directly increasing the risk of diseases such as hypertension and diabetes, thereby exacerbating the CKD burde (51). As a developing country, Mauritius has a well-developed medical system in urban areas; however, medical resources may be unevenly distributed in rural and remote regions. The experience of Mauritius demonstrates that in resource-limited countries or regions, improving medical facilities and resource allocation—especially strengthening primary healthcare services—can more effectively address the high burden of CKD (52). Increasing investment in community health services and improving public health system coverage will help intervene in chronic diseases at an early stage. Other developing countries and regions should learn from Mauritius’ experience by strengthening early screening, health education, and lifestyle interventions, while improving medical resource allocation and establishing a more comprehensive social support system to effectively address CKD challenges.

Owing to varying population sizes, countries with larger populations tend to report higher absolute DALY values compared to those with smaller populations. According to the world distribution map of ASDR, developed regions like Europe, North America, and Australia exhibit a lower CKD-related burden, whereas many African and some South American countries, along with certain Middle Eastern and Southeast Asian regions, display a higher burden of CKD. Comprehensive public health policies are crucial for treating CKD and preventing ESRD. These include implementing early diagnosis and treatment programs, advocating for and educating high-risk populations, promoting renal protective treatments, and effectively controlling risk factors like elevated systolic blood pressure and blood sugar (53). Given the notable differences in age, gender, and related ASMR and ASDR among CKD patients, developing targeted disease prevention, diagnosis, and treatment strategies is essential, rather than relying on one-size-fits-all guidance. This tailored approach is crucial for addressing and alleviating the challenges posed by CKD.

The SDI is a comprehensive measure of a country or region’s social and demographic development. SDI is commonly used to assess the distribution trends of diseases and mortality across various development levels in countries or regions, thus highlighting health inequalities. The findings of this study indicated that high-income developed regions with higher SDIs, such as Europe, North America, and Australia, exhibited a lower burden of CKD disease. Conversely, underdeveloped regions like Africa, Southeast Asia, and Latin America showed relatively high ASDR values, corresponding to a greater CKD burden. Additionally, the CKD burden was higher in men than in women across nearly all regions, with the disparity between men and women being less pronounced in developed areas compared to underdeveloped ones. CKD trends vary across regions with different SDI. In low-income countries, the CKD mortality rate may be higher due to the lack of early diagnosis and treatment; whereas in high-income countries, the mortality rate has been controlled through better medical resources and management, but prevalence remains influenced by unhealthy lifestyles (34). The burden of CKD in high-income countries is primarily driven by aging, whereas low- and middle-income countries are more influenced by the prevalence of chronic diseases and limited medical resources (54). These results suggested that the global CKD burden was more severe in low-income countries, where primary healthcare systems were less advanced, hindering effective early screening, prevention, diagnosis, and management of chronic diseases (55). Therefore, we suggest that future policy-level support should focus on strengthening early screening, public health education, and the development of medical infrastructure in low- and middle-income countries to effectively mitigate the rising burden of CKD. Specifically, in low-SDI areas, promoting health education in schools and communities to raise public awareness of CKD risks could be one of the most effective strategies to reduce its prevalence (56). Promoting government investment in improving medical infrastructure and expanding basic health screening, particularly in remote areas, along with establishing more diagnostic and treatment facilities and enhancing the accessibility of medical services, can significantly reduce CKD mortality rates. By comprehensively implementing financial support, technical cooperation, public health education, and preventive measures, the capacity for CKD management in low-income countries is expected to improve substantially, thereby alleviating the global burden of CKD.

It is crucial to monitor the prevalence and predict trends of chronic diseases such as CKD. According to the ARIMA model, CKD prevalence is projected to reach 11.7% by 2029, and the mortality rate to 17.1 per 100,000 (2, 57). Although the ARIMA model predicts an upward trend in CKD based on current trends, global and regional interventions could significantly alter this trajectory. First, actively promoting early screening and intervention for underlying conditions such as diabetes and hypertension could effectively slow CKD progression, potentially influencing the ARIMA model’s predictions and curbing the upward trend in CKD. Health education programs, promoting healthy diets (e.g., reducing salt and sugar intake and increasing dietary fiber), and encouraging physical activity can help control chronic diseases. This trend could result in a reduction in the predicted increase in CKD incidence and mortality, as forecasted by the ARIMA model. This trend could result in a reduction in the predicted increase in CKD incidence and mortality, as forecasted by the ARIMA model. Implementing these policies could improve the treatment and management of CKD patients worldwide, thereby altering the ARIMA model’s prediction trend (58).

As of 2017, GBD data indicated that the global number of CKD patients surpassed those with diabetes, osteoarthritis, COPD, asthma, or depression (59). Regrettably, both public health departments and the general public in many regions give less attention to kidney health compared to hypertension, diabetes, and cardiovascular diseases. The significant disparity between the high prevalence and low awareness of CKD may contribute to the ongoing rise in mortality rates in recent years. These findings reveal that age-adjusted prevalence and mortality rates for CKD consistently exhibited an upward trend from 1990 to 2021. This upward trend is expected to persist over the next decade. Consequently, it is essential to implement a comprehensive strategy that includes disease prevention at the primary healthcare level, targeted CKD screening for elderly and high-risk groups, and advanced training for healthcare professionals to lessen the CKD burden and improve patient outcomes. In CKD management, the efficient allocation of resources is critical for reducing the disease burden and improving treatment outcomes. The high incidence and progression of CKD are often closely associated with preventable diseases, such as hypertension and diabetes. Preventive strategies can not only reduce the incidence of CKD, but also alleviate the strain on medical resources.Physical activity is one of the most effective strategies for preventing CKD (60). Moderate aerobic exercise, resistance training, and other forms of physical activity may benefit CKD patients by improving cardiovascular health, blood sugar control, weight management, and muscle strength, thus alleviating CKD symptoms and delaying disease progression (61). Therefore, governments and public health agencies can promote active lifestyles through various means, including organizing community fitness programs, constructing public sports facilities, and conducting health education initiatives to encourage exercise and improve health. Through these public health initiatives, we can effectively raise awareness of exercise and promote a healthy lifestyle across society, thereby slowing the incidence and progression of CKD. In addition to prevention, early diagnosis and intervention in patients with CKD can significantly slow disease progression, preserve kidney function, and prevent the progression to end-stage renal disease (ESRD). Additionally, differentiated resource allocation based on the CKD burden in different regions and populations, to ensure the efficient use of resources, is essential. More importantly, governments and policymakers can enhance CKD management, reduce the medical burden on patients, and slow the rising global CKD burden by increasing support for public health policies, adjusting medical insurance, advocating global cooperation, developing and applying new technologies, and introducing novel drugs and therapies.

This study had several limitations that must have been acknowledged. Firstly, the quality and availability of data sources varied across countries and regions, potentially affecting the accuracy of our data analysis. For instance, in some low- and middle-income countries, unreliable epidemiological data and underreporting of CKD cases could have resulted in an underestimation of the actual disease burden. Secondly, the GBD methodology depended on various assumptions and modeling techniques, which could have introduced uncertainties in the estimation process. Although the GBD study employed rigorous statistical methods to address these uncertainties, the results should be considered the best estimates based on the current evidence. Finally, we advocated for the continued use of diverse analytical methods to validate the findings of this study.

This study utilized the GBD2021 database to conduct a comprehensive analysis of the epidemiological characteristics of CKD across 204 countries and regions from 1990 to 2021, exploring the relationships between CKD and factors such as age, gender, and the SDI. In addition, this study assessed worldwide CKD prevalence, mortality, DALYs, and DALY rates, examining gender disparities, the impact of SDI on CKD burden, and analyzing epidemic trends across various countries and regions. Additionally, the study leveraged historical data to forecast global CKD trends from 2022 to 2032. Given the anticipated increase in CKD prevalence and mortality over the next decade, it is imperative to expand screening coverage, enhance monitoring, and refine risk factor screening programs to boost CKD diagnostic efficiency, while fortifying global health management and promoting proactive health concepts to address the escalating CKD disease burden.

## 5 Conclusion

A rising global disease burden of CKD. In this study, regarding gender, the prevalence, mortality, and DALY rates for men are generally higher than for women. As SDI increases, CKD DALY rates begin to decline, particularly in high SDI regions, where the disease burden is significantly lower than in low SDI areas. Furthermore, age-standardized CKD prevalence and mortality rates have continued to rise from 1990 to 2021, with this upward trend expected to persist beyond 2022.

## Data Availability

The original contributions presented in the study are included in the article/supplementary material. Further inquiries can be directed to the corresponding author.
